# Effectiveness of Nanoparticle-Based Acidulated Phosphate Fluoride (APF) Gel on Surface Enamel Fluoride Uptake: an Interventional Study

**DOI:** 10.30476/DENTJODS.2021.87895.1295

**Published:** 2022-03

**Authors:** Anusha Raghavan, Aparna Sukumaran, Madan Kumar Parangimalai Diwakar

**Affiliations:** 1 Dept. of Public Health Dentistry, 2/102,East coast road, Uthandi, Chennai-600119, Tamil Nadu, India; 2 Dept. of Public Health Dentistry, Ragas Dental College and Hospital, Tamil Nadu, India

**Keywords:** APF gel, Biopsy, Enamel fluoride, Nanoparticles

## Abstract

**Statement of the Problem:**

Despite topical fluoride being used for over 50 years in caries prevention, its complete potential in terms of formation of fluorapatite enamel and prolonged surface retention has not been harnessed.

**Purpose:**

This study aimed to assess the effectiveness of nanoparticle based acidulated phosphate fluoride (APF) gel on surface enamel fluoride uptake through split mouth design over a period of six months, on patients undergoing orthodontic treatment and indicated for bilateral extraction of premolars on at least one of the arch.

**Materials and Method:**

A split mouth non randomized clinical trial was performed. Each of the 30 participants received one application of 4 minutes duration of both the intervention: Right half of the mouth received nanoparticle based APF gel, and left half of the mouth received conventional APF gel (16 Oz Pascal Corp.; strawberry flavor). Bilateral acid etch biopsy of enamel surface was take at 3 intervals- baseline,24 hours and 30 days. Using 1ml of 0.5M perchloric acid, acid etch enamel biopsy was taken for all the study subjects at 3 intervals of time (baseline, 24 hours and 30 days), bilaterally on the buccal and palatal/lingual surface of maxillary or mandibular premolars indicated for extraction, using 1 (l of 0.5M perchloric acid. After the premolars were extracted, scanning electron microscope (SEM) analysis was done to determine the surface characteristics of enamel in both groups.

**Results:**

Overall, both fluoride uptake and depth of biopsy remained significant even after controlling for the covariates (time, group) individually and simultaneously (*p*< 0.05). Further analysis showed that fluoride uptake was significantly increased and biopsy depth significantly decreased in the nanoparticle based APF gel group at 24 hour and 30 day evaluation respectively.

**Conclusion:**

By reducing the size of sodium fluoride to increase the surface enamel uptake, our results support the use of this top down approach as a promising strategy for effective delivery of topical fluorides. This highlights that the top down approach of nanoscience to reduce the size active compound sodium fluoride has increased the uptake and retention of surface enamel fluoride.

## Introduction

Over 50 decades, fluorides have been the cornerstone in the prevention of dental caries, and have proven benefits on both topical and systemic supplementation [ 1 ]. The term ‘*topically applied fluoride*’ refers to those delivery systems which provide fluoride to exposed surfaces of the dentition, at elevated concentrations, for a local protective effect and are therefore not intended for ingestion [ 2 ]. In the present scenario of evidence based practice, the American Dental Association [ 3 ] substantiates the efficacy of the acidulated phosphate fluoride (APF) gel as a one of the cost effective means among the various professionally applied topical fluoride (PATF) agents, next to fluoride varnish [ 3 ].

Despite the proven effectiveness of fluoride gels in caries prevention, there still remains a gap between the quantity of fluoride supplemented and the quantity taken up by the enamel surface which is in turn responsible for the prevention of demineralization [ 4 ]. In order to overcome this, alternative strategies have been tried to improve the fluoride uptake like altering the temperature of the topical fluoride agent, surface treatment of the enamel with phosphoric acid, prophylaxis of teeth before application, combining chelating metallic ions and laser irradiation of the enamel surface [ 2 ].

Although numerous improvements have been made, economic level implications of these when applied on a community wide basis is questionable. Technological advancements such as the use of nanoscience, works on particles of dimension one hundredth of a micrometer [ 5 ]. First described by Dr. Richard P Feyman in 1959, nanotechnology has made a paradigm shift in all the areas of dentistry from prevention to restorative and surgical materials[ 5 ]. However, in preventive dentistry, it has been largely restricted to the alteration of the oral biofilm by addition of metallic nanoparticles to existing fluoride varnishes, remineralizing agents like casein phosphate protein or restorative materials like dentin bonding agents, composites, and cements [ 6 ].

Moreover, many authors have suggested that the improvement in fluoride therapy should be such that the formulation results in increased concentrations of permanently bound fluoride [ 3 , 7 - 8 ]. One other possible way, which is still unexplored in improving the enamel uptake of fluoride from gels, is altering the particle size of the active component which is sodium fluoride (NaF). This sodium fluoride compound has an octahedral lattice of 462pm with each particle measuring approximately 99.47µm in diameter. Literature evidence shows that over 90% of this fluoride is freely available as calcium fluoride, which predominantly dissolves with only a small fraction being available in bound form of fluorapatite within the enamel crystals [ 2 ].

*In vitro* and *in vivo* studies using APF gel have reported an immediate increase in fluoride uptake following topical application, which gradually decreased with increasing depth or time of measurement [ 7 , 8 ]. An indirect method of this surface enamel fluoride uptake was through measuring the surface microhardness of the extracted tooth samples. An average increase of 40-60% in the hardness values, 24 hours following the application of APF gel has been demonstrated from *in vitro*, in situ and *ex vivo* models by researchers across the globe in both deciduous and permanent dentitions [ 9 - 10 ]. Our previous study on extracted teeth also showed similar results at 24 hour evaluation, which gradually decreased during the one month observation period [ 11 ]. However, the inability of these models to simulate oral conditions implies the need for better *in vivo* studies to assess the fluoride uptake from topical gels by the enamel surface. Hence, it was hypothesized that the reduced particle size of sodium fluoride and incorporation in freshly prepared APF gel will alter the fluoride uptake compared to the conventional gel, which may in turn affect the fluoride retention on the enamel surface.

## Materials and Method

### Trial description

**Figure 1:**
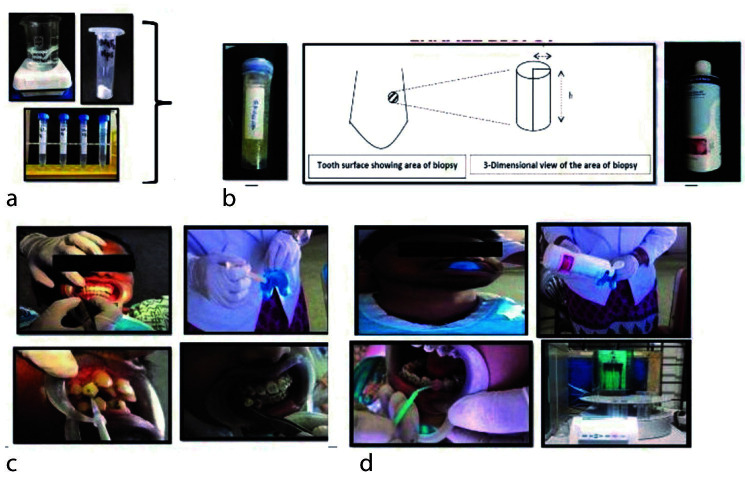
**a:** Preparation of nanoparticle based gel, **b:** The schematic representation of the enamel biopsy procedure, **c:** The clinical photograph of how the biopsy was performed at baseline, 24 hours and 30 days, **d:** The application of gels in their respective sites and analysis.

A bilingual written informed consent was obtained from the participants of age above 18 years and parental consent was obtained for participants of age 15 to 18 years. Permission to conduct the study was obtained from the Head of the institution; Head of the Department, Department of Orthodontics, Ragas Dental College and Hospital and the Project Head, Hubert Enviro Care Solution Private Limited, Vadapalani, Chennai. This was a split mouth active controlled, phase II interventional study where the nanoparticle based APF gel was prepared [ 12 ] and applied to the right half of the patients’ mouth while the conventional gel (16 Oz Pascal Corp.; strawberry flavor ) was applied to the left half of the mouth. Fluoride uptake of enamel was assessed by acid etch biopsy[ 13 ] using 0.5M perchloric acid at time intervals of baseline, 24 hours and 30 days after intervention. The study was conducted over a period of 6 months, from May to November 2018 (Figure 1).

### Participants

Only participants who reported to the Department of Orthodontics for correction of malalignment, between age 15-30 years, who gave a written consent to participate in this study and those indicated for extraction of premolars bilaterally in at least one arch for orthodontic correction due to any one of the following reasons were included in the study. The reasons for orthodontic treatment included discrepancy between tooth material and arch length of more than 5mm, Class II division 1 arch relation on Skeletal I base with mild mandibular crowding, correction of bimaxillary protrusion, mild class III arch relationship with mild crowding in maxillary arch, and ectopic eruption/ presence of impacted canines.

In addition, it was ensured that the participants were permanent residents of Chennai where the optimal fluoride levels in the drinking water is restricted to 1 ppm were. All participants who had systemic disorders and/ or who were under medications, who were indicated for orthodontic correction without extraction of premolar teeth, extraction of any teeth other than premolars or any other method of correction of teeth were excluded. Other reasons for exclusion were the presence of developmental defects of enamel, caries or restoration present on premolars and those who developed any form of acute dental problems during the study period.

### Sample size

The sample size for each group was calculated using the G power statistical software. The mean values from our previous study [ 11 ] were taken into consideration for sample size estimation as microhardness is assumed to be proportional to the amount of fluoride uptake.

**Figure 2:**
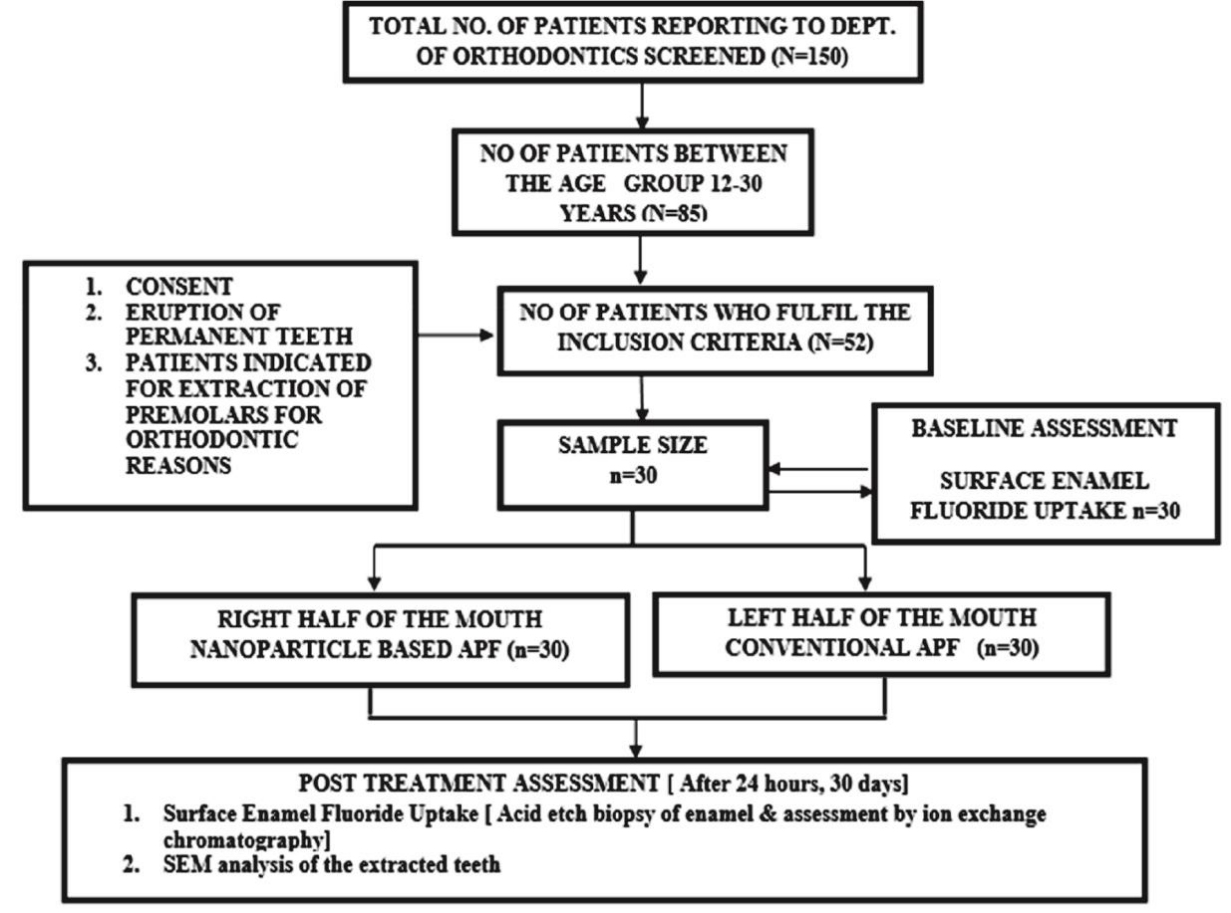
Flowchart illustrating the methodology of the study

The power of the study was kept at 80%, with an alpha error of 0.5 and the estimates were calculated to 95 % confidence interval. In order to account for loss to follow up, during the course of the study and based on the availability, 30 participants were included in the study. Figure 2 represents the flow of participants through the study.

### Calibration and Biopsy

A single calibrated investigator performed the acid etch biopsy in order to avoid the variations in the amount of biopsy sample collected from the study participants. A type III clinical examination was carried out in the dental chair at the Department of Public Health Dentistry to ensure the oral hygiene is good and there are no clinical visible active carious lesions. The patient was kept in semi-supine position. The premolars indicated for bilateral extraction at a later date were used for fluoride assessment.

The collected biopsy samples were transported in sealed individual pouches within a span of 10 days to the laboratory. Each sample was neutralized by adding 1N NaOH drop by drop. One milliliter of the neutralized solution was then made up to 10ml with distilled water. From this stock solution 1ml was taken for analysis. The biopsy solution was analyzed for fluoride and phosphate ions. The mass of enamel biopsied was calculated based on the assumption that enamel contains 17.5 % phosphate by weight. The depth of the biopsy was calculated based on the following assumptions: (i) density of enamel is 2.95g/cm^3^; (ii) the geometry of the biopsied site was a cylinder. The same procedure was repeated in the subsequent analysis of biopsy samples after 24 hours and 30 days. The amount of fluoride taken up by the enamel surface was computed using the formula:

Mass of enamel biopsied=Mass of PO4 biopsied/ 0.175

Depth of biopsy= Mass of enamel biopsied÷[(Density of enamel)*(Biopsy surface area)]

$=\frac{(\mathrm{Praw}-0.37)*3*1000}{3.14*\mathrm{depth}*0.175*2950/1000}$

$F-\mathrm{Content in ppm}=\frac{(\mathrm{Fraw}-0.1)*1000000}{3.14*\mathrm{depth}*2950/1000}$

Fluoride estimation was done using 850 Professional IC- AnCat- MCS (2.850.3030). At a time only one gel was applied in both the arches using foam trays of appropriate size. During each application the premolar teeth on the opposite quadrant were covered with Teflon tape to avoid cross-over effect from the tray. A time span of 30 minutes was maintained between the application on the right and left halves of the mouth. During each application, the subjects were asked to tilt their head forward by 45º to prevent accidental ingestion of fluoride and a high volume suction was used to aspirate the excess fluoride during each application.

Similar to baseline assessment, procedure for acid etch biopsy of enamel was performed on the buccal or palatal/ lingual surface of the respective premolars on either side. In order to avoid re etching of the same biopsy site again, after 24 hours the biopsy was done on the distal half of the buccal surface of the premolars and after 30 days, the biopsy was done on the palatal/ lingual surface of the corresponding tooth. Also after each biopsy, the corresponding test or control APF gel was applied with the help of microbrush on the biopsied area for 4 minutes (nanoparticle-based gel on the right side and conventional gel on the left side).

Furthermore, the extraction of the premolars on either side was done atraumatically under aseptic conditions at two intervals of time using closed method with teeth on one side being extracted at a time. The extraction was carried out within 30 to 60 days post the final evaluation. The extracted teeth were collected separately in two different containers prefilled with 5ml formalin for 24 hours. Afterwards, two teeth were randomly selected from each group, washed under running water and air dried. The root portion of the teeth were sectioned and removed and the crown was sliced longitudinally with the help of carborundum disc on a slow speed hand piece. The sectioned portion was mounted on circular resin base of 2cm diameter, made of poly methyl methacrylate. The mounted specimens were observed under scanning electron microscope.

Data obtained were compiled systematically in Microsoft Excel spreadsheet. Statistical analyses were performed using Statistical package for Social Sciences software (IBM Corp. Released 2011. IBM SPSS Statistics for Windows, Version 20.0. Armonk, NY: IBM Corp). Normality of the data was assessed using Kolmo-gorov-Smirnov test and Shapiro- Wilk numerical test. Due to the unequal difference in between the 3 time points of estimation, interaction effect was assessed using generalized estimating equation (GEE). Depending upon the significance, appropriate parametric statistical tests were chosen p value of < 0.05 was considered to be significant.

## Results

The mean age of the study population was 22.27±4.5 years with 12 males (40%) and 18 females (60%). Both individual and combined effects of covariates on the fluoride uptake and biopsy depth as independent variables are given in Tables 1 and 2. On the whole, fluoride uptake and depth of biopsy showed significant difference when controlled for the covariates individually and simultaneously (*p*≤0.05). 

**Table 1:** Generalized estimating equation with fluoride uptake as independent variable controlling for group, time and simultaneously for group and time

Parameter	B	Std. Error	95% Wald CI		Hypothesis Test		
			Lower	Upper	Wald Chi-Square	df	*p* Value
Group	1874.929	144.6119	1591.495	2158.363	168.098	1	<0.001*
Time	117.174	20.7108	76.582	157.767	32.009	1	<0.001*
Group * Time	-89.124	12.5363	-113.695	-64.554	50.542	1	<0.001*
(Scale)	5498595.4						

Dependent Variable: Fluoride uptake

Model: Group, Time, Group * Time

* *p*< 0.05 indicates significance

**Table 2:** Generalized estimating equation with depth of biopsy as independent variable controlling for group, time and simultaneously for group and time

Parameter	B	Std. Error	95% Wald CI		Hypothesis Test		
			Lower	Upper	Wald Chi-Square	df	*p* Value
Group	42.405	1.4835	39.497	45.312	817.039	1	<0.001*
Time	2.495	.0313	2.434	2.556	6372.593	1	<0.001*
Group * Time	-1.509	.0569	-1.621	-1.397	702.610	1	<0.001*
(Scale)	313.365						

Dependent Variable: Biopsy depth

Model: Group, Time, Group * Time

* *p*<0.05 indicates significance

Table 3 represents the distribution of mean fluoride uptake at the baseline, 24 hours and 30days, which showed a significant difference after 24 hours (*p*= 0.046). Intragroup comparison showed a significant decrease in the mean fluoride uptake on pairwise comparison of all 3 time points.

**Table 3:** Mean difference in Fluoride uptake between nanoparticle based acidulated phosphate fluoride gel and conventional acidulated phosphate fluoride gel (both between the groups and within group comparison)

Time of Testing	Nano-Based Gel (n=30)	Conventional APF GEL (n=30)	*p* Value
BASELINE	1487.69±826.8†,¶	1509.39±844.83§,∞	0.920
AT 24 hrs	5495.25±2171.12†,‡	4317.86±2294.33§,●	0.046*
After 30 days	2492.64 ± 1491.49 ‡,¶	1944.25 ± 1087.67●,∞	0.109

* *p* value <0.05 indicates significance

†,‡,¶ indicates pair wise comparison within nano-based APF gel group at baseline, 24hrs and 30 days with significance at the level of *p*< 0.001

§,●,∞ indicates pair wise comparison within conventional APF gel group at baseline, 24hrs and 30 days with significance at the level of *p*< 0.01

Table 4 represents the distribution of mean depth of enamel biopsy, which showed a significant difference at the 30 days evaluation (*p*= 0.046). Intragroup comparison group showed that there was an average significant decrease in the biopsy depth from 71.15±3.18µm at the baseline to about 69.33± 2.11µm in nanoparticle based gel group during the 30 days of evaluation (*p*≤0.05) while the conventional group did not show any difference.

**Table 4:** Mean difference in the depth of enamel biopsy between nanoparticle based acidulated phosphate fluoride gel and conventional acidulated phosphate fluoride gel (both between the groups and within group comparison)

Time of Testing	Nano-Based Gel (n=30)	Conventional APF Gel (n=30)	*p* Value
BASELINE	71.15 ± 3.18†,¶	70.92 ± 2.95§	0.767
AT 24 hrs	70.18 ± 2.92†,‡	70.31 ± 2.93§	0.858
After 30 days	69.33 ± 2.11 ‡,¶	70.45 ± 2.15	0.046*

* *p* value <0.05 indicates significance

†,‡,¶ indicates pair wise comparison within nano-based APF gel group at baseline, 24hrs and 30 days with significance at *p*= 0.00,0.027 and 0.00 respectively

§ indicates comparison of conventional APF gel group at baseline and 24 hours significance at the level of *p*= 0.002

**Figure 3:**
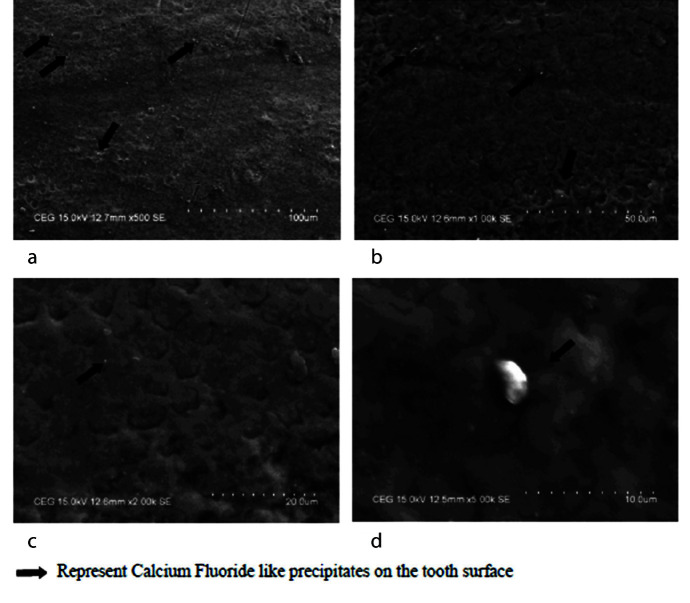
Scanning electron microscope images of tooth surface from nanoparticle based acidulated phosphate fluoride gel group at 500x, 1000x, 2000x and 5000x magnifications

**Figure 4:**
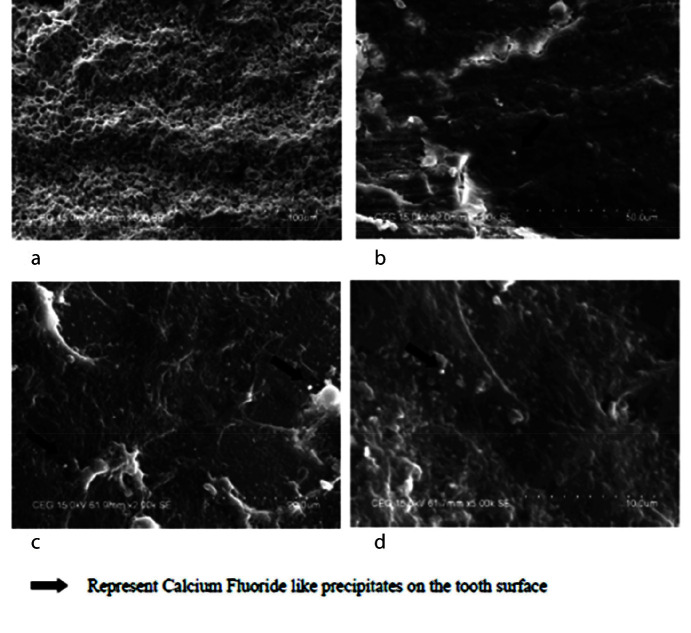
Scanning electron microscope images of tooth surface from conventional based acidulated phosphate fluoride gel group at 500x, 1000x, 2000x and 5000x magnifications

Scanning electron microscope (SEM) analysis of the extracted teeth samples showed calcium fluoride like precipitates deposited on the tooth surface of samples from both the groups. However, the prismatic nature of the enamel surface was better retained in the nanoparticle based APF gel group compared to the conventional group which was similar to an etched surface (Figures 3-4).

**Figure 5:**
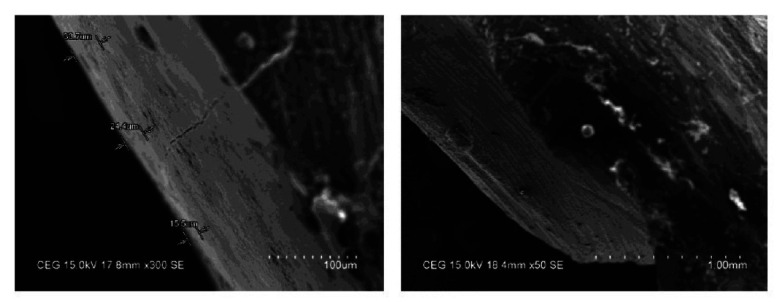
Longitudinal section of tooth sample from nanoparticle based and conventional acidulated phosphate fluoride gel group

Examination of the longitudinal section of the extracted tooth samples under SEM showed the presence of a glossy layer above the enamel surface in the nanoparticle based group (Figure 5a). However, no similar layer was seen on the sample from the conventional gel group (Figure 5b).

## Discussion

Topical fluoride preparations have evolved as additional means to deliver substantially higher concentrations of fluoride to improve the quality of the enamel surface and amongst the PATFs, APF gel has been time tested for its cariostatic action [ 14 ]. The topical fluorides are mainly available in two major chemical forms inorganic and organic fluoride [ 2 ]. The anticariogenic action of topical fluorides depends on the solubility of the fluoride containing compound and its adhesion to the tooth surface. This anticariogenic property of inorganic fluorides are predominantly due to decrease in the enamel solubility, enhanced remineralization and antienzymatic property by formation of fluorapatite crystals, improving the crystal growth, stabilizing the crystal structure, affecting the enzymatic glycolytic pathway, sugar transport and intracellular pH homeostasis of bacteria respectively [ 15 ]. When analyzed in terms of bound and free fluoride which are essential in determining the anticariogenic properties, inorganic fluorides perform better by providing high concentrations of free fluoride [ 7 ]. Amongst these, APF which contains sodium fluoride mainly acts through the formation of calcium fluoride precipitates and fluorapatite by reacting with calcium present in the enamel crystals.

The higher concentration and low pH of APF, results in the formation of large quantities of calcium fluoride (CaF_2_) and dicalcium phosphate dihydrate (DCPD). The acidic pH further favors the uptake of fluoride ions by enamel surface, by slight dissolution of the enamel minerals in the superficial layers [ 1 ]. A transitional phase is thus established, during which almost 90% of the fluoride ions were incorporated on the enamel surface. This results in a transient phase in the period initially after topical application where there are high levels of fluoride seen which accounts for almost 90% of the fluoride incorporated on the tooth surface [ 1 ]. As time elapses, this CaF_2_ dissolves rapidly in the oral environment leaving behind only a small fraction of firmly bound fluoride in the form of fluorapatite. Hence, an effective topical fluoride regimen would be the one which results in a relatively higher concentration of permanently bound fluoride on the enamel surface. The presence of this free or available fluoride is evaluated in terms of fluoride uptake from the tooth surface, and is restricted to the first few layers of enamel [ 1 - 2 ].

The effective mode of delivery of the APF was assessed at 15 minutes, 16 days showed similar results in both the groups, concluding that, both solution and gel, were equivalent in their effectiveness [ 16 ]. Time dependent fluoride acquisition from APF gel was tested in laboratory and *in vivo* conditions [ 17 - 18 ]. The authors concluded that the fluoride concentrations in the enamel was maintained high during the treatment periods and sustained for an average of 2 to 10 months after which the levels stabilized at 1600 ppm [ 17 - 18 ]. They found that fluoride uptake significantly increased with increase in the contact with topical agent and recommended professional application time of 4 minutes.

Few authors concluded that presence of unclean teeth did not affect fluoride uptake or the caries inhibitory properties of fluoride but only the time and labor intensity required per patient was drastically reduced in the absence of prophylaxis procedure [ 19 - 21 ]. This was effectively found in our study, where the maximum time taken for APF gel application and the enamel biopsy together was less than 10 minutes for each half of the mouth, with an interval of 30 minutes in between, after which the patient was asked to rinse thoroughly. This ensured that cross over effect of one agent over the other is minimized.

The uptake and retention of fluoride on the enamel surface had been assessed through a variety of methods amongst which many modifications have been tried for the acid biopsy technique predominantly differing in terms of with/ without the use of burs (silicon carbide, felt burs, impregnated burs) or the type and concentration of acid used (hydrochloric, perchloric acid)[ 7 , 13 , 16 - 26 ]. In our present study, the field biopsy technique [ 13 ] was used as it did not involve any invasive procedure and was performed only on specific demarcated area and not the whole teeth.

Applications of laser and cold atmospheric plasma have also been tried in the recent past in combination with APF gel have also showed promising results in increasing fluoride uptake [ 22 - 24 ]. In line with these advancements, a commendable stride has been made with the introduction of nanoscience in healthcare [ 6 ]. A randomized, double blinded *in-situ* trial found that the combination of nanohydroxyapatite crystals with sodium fluoride (NaF) on dentin remineralization improved fluoride delivery into early dentinal lesions and prevented demineralization [ 25 ].

Our present study showed an overall increase in the fluoride uptake in both groups following APF gel application. However, the average increase in the nanoparticle based group was higher than the control at 24 hour evaluation which gradually returned close to baseline at the end of 30 days in both the groups. This was similar to the mean fluoride concentration of 6520±407 ppm, 15 minutes after gel application [ 16 ].

On comparing the biopsy depth at different time points, our study showed a minimal but gradual significant decrease from 71.15±3.18µm to 69.33±2.11µm in the nanoparticle based group whereas, similar estimation in the conventional group was not significant.

Clinical estimation of the depth of enamel biopsy has been done in literature earlier [ 16 ]. However, the above estimations were done on maxillary incisors of children of 8 to 12 years which is different from our study population.

Similar observations in extracted teeth samples in in-situ conditions for APF was found to be around 2.3 to 9.1µ in the 1 to 5 layers of enamel over an etching period of 120 seconds [ 26 ]. This could also be not considered equivalent as the assessments were done on extracted teeth samples mounted on prosthetic appliances which was worn by 12 volunteers.

*In vitro* experiment on 528 enamel specimens to assess uptake and retention of APF, thixotropic and pluoronic gel showed an average presence of 6929±6091 ppm, 5913±10,135ppm and 3566±1463ppm of fluoride respectively immediately at 24 hours, 7 and 28 days evaluation with a mean biopsy depth of 3.02±1.19µm for the APF group [ 27 ]. A cumulative etching depth of 54µm after an etching period of 20 minutes with 0.1M HCl acid has also been reported [ 28 ].

Further analysis of the extracted teeth revealed deposition of calcium fluoride like precipitates on the surfaces of specimens from both the groups, similar to those in the literature [ 29 - 31 ]. However, the surfaces of the specimen in the nanoparticle based APF group showed finer characteristics in terms of absence of loss of prismatic structure which was not established in the conventional group. This was similar to the findings reported by few authors who reported that topical application of APF resulted in loss of minerals on the tooth surface due to acidic pH and created lacunae [ 17 - 18 , 27 ].

On the whole, there is very little evidence comparing the uptake and retention of fluoride from APF gels in clinical conditions of which none of them are from the recent past, thus preventing the possibility of direct comparison to the current results. The earliest evidence of assessing minerals in the tooth surface dated back to 1968 when it performed the procedure on military personnel to demonstrate the usefulness of this method [ 32 ].

To the knowledge of the authors, this was the first attempt to modify the particulate size of the sodium fluoride, the principal component of APF in order to enhance fluoride fixation in enamel. The major strengths include the use of split mouth design which facilitated simultaneous evaluation of 2 different interventions. This was a phase 2 trial done after prior evaluation of the new agent in *in vitro* conditions with the clinical evaluation supported by microscopic evaluation of the extracted teeth at the end of study. However, differences in the fluoride levels on varied areas of the tooth surface and between maxillary and mandibular teeth were not taken into account, no attempt was taken to randomize the participants and the effect of fluoride from other topical agents like dentifrices, mouthrinses, food and beverages were not considered.

## Conclusion

The present study highlights the effective application of nanotechnology in preventive dentistry by modifying the particle size of sodium fluoride compound in APF gel. Further evaluation of this nanoparticle based gel in terms of maximum achievable reduction in particle size, alteration in duration of application, frequency of application and use of chelating compounds should be done to substantiate its long-term effectiveness.

## Acknowledgement

The authors thank Avanz Bio Lab Private Limited, East Tambaram, for their support in the synthesis of sodium fluoride nanoparticles, Mr.Ravi and Mr. Mohan from Hubert Enviro care Pvt Ltd, Vadapalani for their technical assistance in fluoride analysis. The authors also take this opportunity to thank Dr.N.R.Krishnaswamy, Professor and Head, Department of Orthodontics, Ragas Dental College and Hospital, Uthandi, Chennai for extending his support in completion of this study. Finally, the authors extend their warm gratitude to all the subjects who participated and were cooperative till the completion of the final evaluation.

This study was started after obtaining ethical clearance from the Institutional Review board and registering in the Clinical Trials Registry- India (CTRI) hosted at the ICMR's National Institute of Medical Statistics (http://nimsicmr.nic.in) (Reference no: REF/2018/05/ 019794; Trial registration no. CTRI/2018/05/013848). This study was conducted in accordance with The Declaration Helsinki.

## Conflict of Interest

The authors declare no competing interest
